# STAYING SINGLE INTO VERY LATE LIFE: LONGITUDINAL ANALYSIS OF NEVER-MARRIED ADULTS

**DOI:** 10.1093/geroni/igad104.2199

**Published:** 2023-12-21

**Authors:** Natasha Nemmers, Peter Martin, Barbra Brottman

**Affiliations:** Wayne State University Institute of Gerontology, Detroit, Michigan, United States; Iowa State University, Ames, Iowa, United States; Iowa State University, Ames, Iowa, United States

## Abstract

With the rise in life expectancy, the phenomenon of singlehood has also increased. However, most studies group unmarried individuals together (i.e., divorced, separated, widowed, never-married), and less is known about lifelong singlehood. This study aims to understand gender and never-married/married status differences in cognition, difficulty in activities of daily living (ADLs), and survival rates. Using the Health and Retirement Study (HRS) of older adults 69 years and older, we conducted longitudinal analyses of health from waves 2-5 and a survival analysis with waves 2-14. The never-married group (n=243) had a higher proportion of racial/ethnic minorities and women than the married group (n=7328) at baseline. Survivorship trajectories by marital group were significantly different: χ2(1) =13.26, p < .001, such that married persons had a better chance of survival than never-married persons. We found significant gender differences in physical activities of daily living (PADLs, F=6.75, p<.001) and instrumental activities of daily living (IADLs, F=9.03, p<.001), indicating that women had greater functional difficulty than men. Regarding marital status, never-married persons had significantly more difficulty in ADLs than married persons only in waves 4 (F=10.22, p<.01) and 5 (F=4.66, p<.05). No cognition differences were found by gender or marital status. Consistent with prior work, these findings demonstrate the protective effect of marriage on mortality, though IADL-difficulty and cognition did not differ. This study will add to the sparse literature on lifelong singlehood. Future studies should consider other relevant covariates, like social network and personality, to inform tailored health interventions and policies for this subpopulation.

